# Peer Review of “Impact of Modifiable Risk Factors on the Occurrence of Cutaneous Leishmaniasis in Diyala, Iraq: Case-Control Study”

**DOI:** 10.2196/31515

**Published:** 2021-07-30

**Authors:** Abdulkareem Ali Hussein Nassar

**Affiliations:** 1 TEPHINET – The Task Force for Global Health Decatur, GA United States

**Keywords:** cutaneous leishmaniasis, outbreak, Iraq, risk factors, risk, disease, infectious disease, disease prevention, prevention

*This is a peer-review report submitted for the paper “Impact of Modifiable Risk Factors on the Occurrence of Cutaneous Leishmaniasis in Diyala, Iraq: Case-Control Study.”*

## Round 1 Review

### General Comments

This paper [1] is suitable for publication by this journal. The importance of this study is that it aims to identify possible risk factors and the impact of removing these factors on reducing the number of cutaneous leishmaniasis cases in Diyala, Iraq, in 2018. It provides evidence-based information to be used for prevention and control measures.

### Specific Comments

Although this paper has a large sample size, it is not clear how the sample size is calculated.

#### Major Comments

There is no major comment.

#### Minor Comments

This paper needs some minor revisions.

##### Abstract

1. Results section: the word “persons” in the sentence “Data from 844 persons (cases=432, 51.2%) persons were analyzed.” is repeated. Therefore, it should be deleted.

2. Results section: I suggest the authors include quantitative results in the Abstract (odds ratios with confidence intervals, etc).

3. I suggest the authors merge the Recommendations under the *Conclusion* section.

##### Introduction

4. The authors explained the abbreviation (CL) for cutaneous leishmaniasis at the beginning of the introduction but sometimes did not use it in the main text.

##### Methods

5. “Further details on why we selected these two districts and how the study was conducted were published elsewhere.” The authors should delete this sentence and explain why the two districts were selected and how the study was conducted in the *Methods* section of this manuscript. Also, delete reference 17.

6. “A total of 866 persons were interviewed within the 717 families visited, 451 cases (292 from Al-Mansuriya District and 159 from Al-Muqdadiya District) and 415 controls (182 Al-Mansuriya District and 233 from Al-Muqdadiya District). However, we excluded 22 persons from the sample due to incomplete information. The final sample size used was 844 persons (cases=432, controls=412).” Although the sample size is large, there is no statistical method to estimate the sample size.

##### Results

7. “Data from 844 persons (cases=432, 51.2%) persons were analyzed.” As mentioned before, the authors must delete the repeated word “persons.”

8. The authors must mention the table in the correct position in the text.

9. Table 1: the number of cases and controls for the “use bed net” and “sleeping habits” variables aren’t similar to the sample size. The authors need to review the numbers and calculate the percentages. Moreover, the authors must explain the total number of cases and controls for the variable “distance of animals from house” in a footnote of instead in the table.

10. Table 2: the attributable fraction for fogging is wrong (10.2 and 28.2). Please change it; the correct fraction is (52.6 and 55.5).

##### Discussion:

11. The authors need more references to compare these findings with previous literature reports.
